# Research Progress of Noise in High-Speed Cutting Machining

**DOI:** 10.3390/s22103851

**Published:** 2022-05-19

**Authors:** Weihua Wei, Yunyue Shang, You Peng, Rui Cong

**Affiliations:** College of Mechanical and Electronic Engineering, Nanjing Forestry University, Nanjing 210037, China; 17306396464@163.com (Y.S.); 18136883601@sina.cn (Y.P.); congrui240859@163.com (R.C.)

**Keywords:** cutting noise, sound source analysis, numerical recognition, noise control, condition monitoring

## Abstract

High-speed cutting technology has become a development trend in the material processing industry. However, high-intensity noise generated during high-speed cutting exerts a potential effect on the processing efficiency, processing accuracy, and product quality of the workpiece; it may even cause hidden safety hazards. To conduct an in-depth study of noise in high-speed cutting machining, this work reviews noise sources, noise collection and numerical recognition, noise control, and condition monitoring based on acoustic signals. First, this article introduces noise sources, noise signal acquisition equipment, and analysis software. It is pointed out that how to accurately classify and recognize the target signal in the complex high-speed machining environment is one of the focuses of scholars’ research. Then, it points out that a computer achieves high accuracy and practicability in signal analysis, processing, and result display. Second, in the aspect of noise signal processing, the characteristics of noise signals are analyzed. It is pointed out that accurately analyzing the characteristics of different noise source signals and adopting appropriate methods for identification and processing are the necessary conditions for effectively controlling and reducing the noise in the process of high-speed cutting. The advantages and applicable fields of artificial intelligence algorithms in processing mixed noise source signals with different frequency characteristics are compared, providing ideas for studying the mechanism of noise generation and the identification of noise sources. Third, in terms of noise control, a detailed overview is provided from the aspects of the treatment of the noise source that contributes the most to the overall noise, the improvement of the tool structure, the optimization of cutting parameters, and the analysis of contact factors between the tool and the workpiece. It provides an effective way for noise control in the process of high-speed cutting. In addition, the application of acoustic signals to condition monitoring is also thoroughly analyzed. The practical application value of condition monitoring based on acoustic signals in high-speed machining is highlighted. Finally, this paper summarizes the positive significance of noise research in high-speed machining and identifies key problems and possible research methods that require further study in the future.

## 1. Introduction

The machinery manufacturing industry is the foundation of the national economy and an important index of the comprehensive national strength of a country [1]. The level of cutting technology determines the quality of industrial products and significantly affects production efficiency and processing costs [2]. High-speed cutting technology is an advanced manufacturing technology that exhibits the advantage of improving cutting speed and feed speed. This technology is regarded as one of the most important and common developments in the cutting and machining field. Compared with traditional machining methods, the high-speed cutting process replaces low cutting speed and large cutting depth with high cutting speed and small cutting depth, and thus, it reduces cutting force, workpiece deformation, and product production cycle. Such modification not only improves cutting accuracy and efficiency, but also reduces production costs. Nowadays, high-speed cutting has attained a major position in the cutting and machining field in industrialized countries and yielded tremendous economic benefits. It has brought a profound technological revolution to machinery manufacturing and will continue to have a significant and far-reaching impact [3,4,5,6,7,8,9,10]. High-speed machining technology, which is a product of this new technology, is widely used in some critical sectors, such as the automobile manufacturing industry, the aerospace industry, and the engineering equipment field [11,12].

During machining, the tools encounter some challenging phenomena, such as chipping edge, breakage, chatter and built-up edge. Therefore, monitoring these phenomena is critical because it can provide a good characterization of the process to maximize production by selecting the best cutting conditions. In addition, the application of monitoring technology has an effective effect on improving production safety conditions [13]. Lauro et al. [14] discussed many monitoring techniques for the cutting process through various literature studies and found that monitoring techniques can be divided into two main categories: direct approach and indirect approach. The direct approach focuses on measuring the actual value of the cutting characteristics, and while it provides a high degree of accuracy, it often requires interrupting machining processes and has a strict demand on the environment, and it is more applicable in the laboratory at the present stage [15,16]. These reasons make it an ineffective tool to detect machining phenomena. The indirect approach relies on the use of empirical correlation to obtain actual values for machining characteristics by processing and analyzing measured signals. Although it provides less accuracy than the direct approach, it has the advantage of the online detection of cutting phenomena. Therefore, the method of predicting machining phenomenon indirectly by analyzing cutting load, current, temperature, vibration and noise is widely used [17,18,19,20].

Noise, as one of the above characteristics, plays an important role in the research of the machining process and has been widely concerned by researchers. Aguilar et al. [21] evaluated surface roughness from friction noise, and a statistical index was used for the artificial neural network (ANN). Especially, the noise signals generated during work are a meaningful indicator for evaluating tool wear [22]. Based on singular spectrum analysis (SSA), information related to tool wear can be extracted from noise signals [23]. Madhusudana et al. [24,25] based on the decision tree (J48 algorithm) technique, extracted a set of discrete wavelet characteristics from the milling cutter noise signal using a discrete wavelet transform (DWT) method for fault diagnosis of the face milling cutter. Lin et al. [26,27] used an artificial neural network to construct an estimation model using data collected from experiments to explore the relationship between tool wear and cutting noise. Pei et al. [22] used a triaxial accelerometer and sound signal sensor to measure the vibration acceleration and processing noise of machine tools, and proposed a new inner diameter saw blade wear evaluation method, which can quickly and timely monitor the inner diameter saw blade wear. Cheng et al. [28] used both current and sound signals to achieve the wear detection of grinding. Qi et al. [29] used the sound signal as one of the parameters to monitor the grinding process, and the improved Mahalanobis distance and CNNs were used to establish the wear diagnosis model. Seemuang et al. [30] developed a tool condition monitoring system that predicts tool wear by measuring noise signals generated by machine spindles. In conclusion, it is a very practical and convenient method to study the machining process by studying the noise generated during the machining process.

In addition, green manufacturing is a significant development trend in mechanical processing manufacturing in this century. Noise not only affects the development of green manufacturing; it is an important factor of an operator’s safety in the machining process [31]. Exposure to high levels of noise rising from machining operations over long periods of time can cause temporary or permanent loss of hearing, the damage is irreversible. High-intensity noise levels can also affect the attention of technicians and interfere with communication in the workshop, leading to an increased risk of accidents [32,33,34]. Robinson et al. [35] conducted audiometric hearing tests on 124 people from 26 companies in Nepal and found that 31% of wood product manufacturers and 44% of wood manufacturers in these companies suffered from hearing loss due to noise levels exceeding the standard level of 85 dB.

In short, the noise in the machining process is a problem that needs special attention in the field of machining. The current work selects noise as the research object and reviews studies conducted on noise source analysis, signal acquisition and numerical identification, and noise control and condition monitoring based on acoustic signals in high-speed machining and other similar fields. This paper has important scientific significance and engineering value for enriching the basic theory of machining noise, and can also provide theoretical guidance for alleviating the process problems of high-intensity noise in the process of high-speed cutting. Moreover, it will provide a reference for researchers engaged in the research of noise in the machining process.

## 2. Noise Source Analysis

Noise generated by materials during high-speed cutting machining is coupled with a variety of different noise sources, and each noise signal typically exhibits varying time and frequency domain characteristics. In actual work, noise mostly originates from the noise emitted by the machine tool, and the cutting noise emitted by the dynamic contact between the workpiece and the tool. The noise source of the machine tool involves its internal structure. Bearings, gears, and motors are all sources of noise in the machine tool [36,37]. Cao et al. [38] performed an idle test on an ordinary spindle and an abnormal spindle with abnormal bearing. They also conducted a systematic study on noise caused by rolling bearings, rotors, and motors, and analyzed the noise source of the spindle at different speeds. Their study found that the rotation of the rotor is the primary factor that causes low-frequency noise, while the main shaft bearing is the major factor that causes high-frequency noise. At low speeds, the noise generated by the bearing contributes considerably to the overall noise. However, when speed exceeds a certain value, the contribution of rotor noise is significantly higher than that of bearing noise. Moreover, the static noise generated by the motor is negligible. Their research is of great significance for reducing the noise of spindle running. The noise of the gearbox in the machine tool is the key component of machine tool noise. Gear noise is caused by various factors, such as gear base pitch error, circumferential pitch error, tooth shape error, tooth direction error, and tooth surface roughness. In addition, other factors that produce gear noise include assembly eccentricity; low contact accuracy; poor parallelism of the shaft; the transverse lines after gear grinding; insufficient rigidity of the shaft, bearing, and support; low rotation accuracy of the bearing; improper clearance; torque fluctuation; gear wear during work; torsional vibration of the shaft system; and balance of the motor and other transmission pairs [39,40,41,42,43,44,45]. Lin [46] pointed out that spectrum analysis technology can efficiently and accurately identify primary noise sources. The primary noise sources that affected the sound pressure level of a lathe were accurately identified by measuring the self-power spectrum of the lathe noise signal and comparing the peak frequency with the noise frequency generated by each pair of gears. The analysis indicated that the frequency doubling noise and gear meshing frequency noise generated by the gear tooth profile error were the primary noise sources.

**Figure 1:**
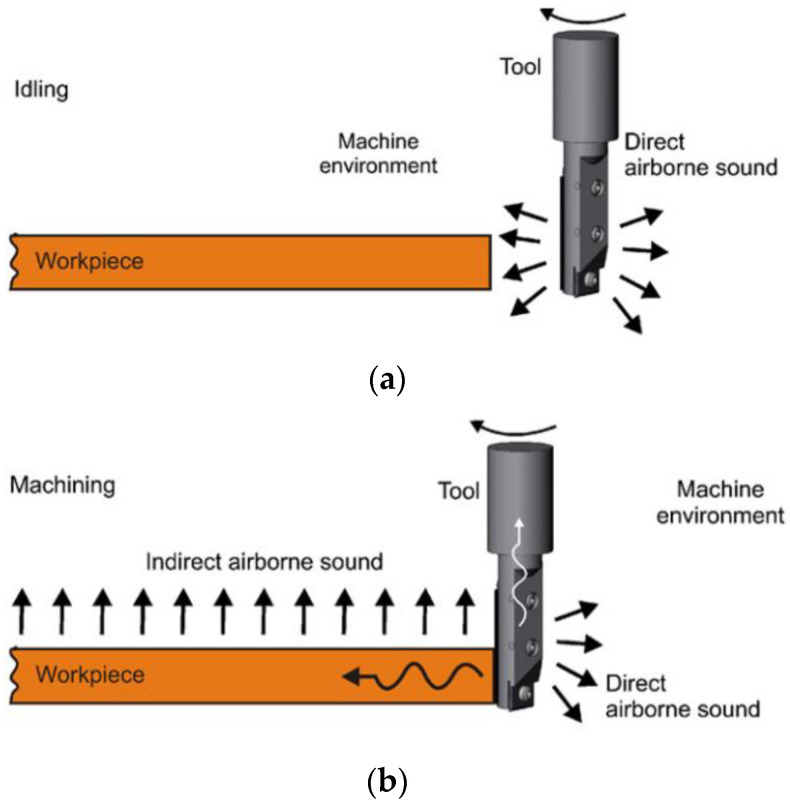
(**a**) Direct airborne noise; (**b**) indirect airborne noise.

Cutting noise has two types. The first type is the process forces that occur during machining. These forces induce direct and indirect airborne noise in the tooth contact zone. Direct airborne noise is composed of the idling sound of the tool and the impulse sound that results from the contact between the tool and the workpiece, as shown in Figure 1a. Indirect airborne noise is induced by the impulse-changing forces that occur during machining, as shown in Figure 1b. The second type is generated by the material displacement and chip removal processes during machining. Both types of noise are emitted directly to the air space of the machine environment [47]. Zhu [48] determined through a micro-milling test analysis of copper that the noise problem in micro-milling is due to the impulse response of the intermittent milling process and the system vibration caused by periodic changes in the cutting force. Ji [49] and Sampath [50] pointed out that noise sources in moving fluids are classified into monopole, dipole, and quadrupole sources. Among them, the dipole sound source is the primary source of the aerodynamic noise of a milling cutter, and broadband noise is the leading reason for overall noise. Moreover, the geometric parameters of a milling cutter also exert an influence on noise generation; among them, the diameter and number of teeth of a milling cutter exhibit a significant effect on the aerodynamic noise of the milling cutter. Hesselbach et al. [47] conducted a high-speed milling experiment and determined that various forces are generated during the machining process, causing the cutter, its connected mechanism, and the workpiece to vibrate and resulting in mechanical noise. Svoren et al. [51] pointed out that the noise at cutting was generated from the transversal oscillation of the circular saw blade of the excitation cutting forces on the teeth. Jin [52] grouped the noise generated by the cutting mechanism of woodworking machine tools into three types: the aerodynamic noise generated by the eddy current of the airflow in the knife groove or the tooth groove, the mechanical noise generated by tool vibration, and the noise caused by different materials and shapes.

The noise of material during high-speed cutting machining originates from a variety of noise sources, and each noise source has its characteristic frequency. A noise signal is a random signal composed of multiple frequency components. The identification and analysis of noise sources during high-speed cutting machining and the accurate analysis of the causes of noise are crucial for subsequent research on noise control.

## 3. Noise Signal Acquisition and Numerical Analysis and Noise Control

### 3.1. Noise Signal Acquisition

Noise is a type of acoustic signal coupled with multiple noise sources. It can be collected by setting the physical channel, noise frequency range, microphone sensitivity, and sampling frequency of equipment, such as a microphone and a sound level meter. The collected noise signal can be converted into a binary digital signal by using a computer sound card. The noise signal can be processed using computer software and transformed into a visual spectrum, enabling us to identify the characteristics of the noise signal accurately. The collection of noise signals is the basis of subsequent research and analysis. Only the timely and accurate collection of noise signals can ensure the correctness of the subsequent research.

**Figure 2:**
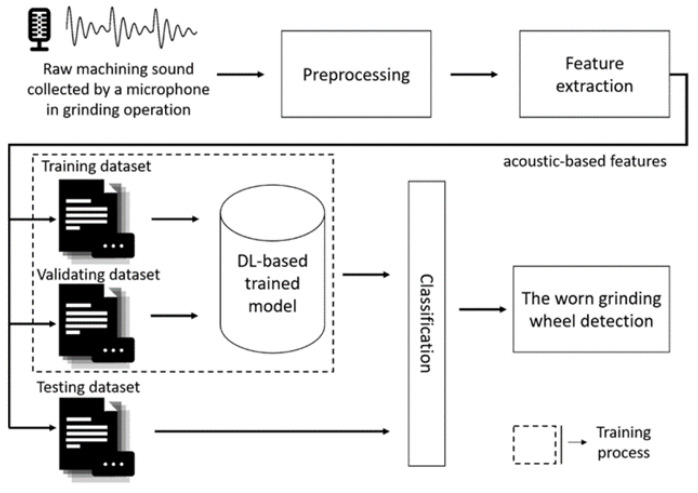
The framework of the proposed intelligent system.

**Figure 3:**
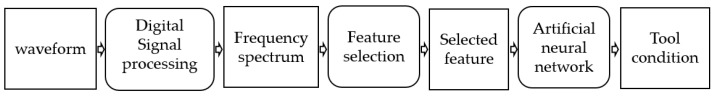
Data flow and data processing in on-line tool wear detection.

**Figure 4:**
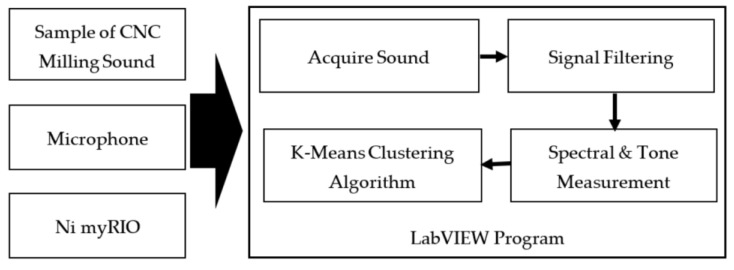
Block diagram of sound detection.

Microphones—the acquisition devices of sound—have the characteristics of low cost, non-disruption to the ongoing operation, and simplicity in sensor placement and setup [53]. Therefore, Microphones are widely used. Cao et al. [38] collected the noise signal of the spindle with three microphones in the experiment. The three microphones used in the experiment were MPA201, with a frequency response between 20 Hz to 20 kHz and a sensitivity of 45.2 mV/Pa and 49.5 mV/Pa. Downey et al. [54] connected a microphone to a Toshiba Satellite laptop via a sound card to record the cutting sound on a lathe. Chen et al. [55] used a microphone to obtain noise values and a spectrum analyzer to analyze the trend of the noise signal. Lee et al. [17] developed an intelligent system based on machining noise and deep learning for grinding wheel condition monitoring to recognize the grinding wheel condition. They embedded a sensor and microphone (sampling rate: 44.1 kHz) in the grinding machine to collect audio signals during the grinding process, and experimental results showed an accuracy rate of 97.44%. Figure 2 shows the framework of the proposed intelligent system for grinding wheel condition monitoring. To collect audio signals during milling tests under varying cutting conditions. Li et al. [56] used three condenser microphones (ECOOPRO EO-200) to collect real-time audio signals from different directions and distances. Prasetyo et al. [57] placed an omni-directional Andoer microphone with a frequency response between 20 Hz to 16 kHz on the tool post to capture audio signal, recorded it on MATLAB at a sampling rate of 44.1 kHz and a sampling size of 1024, and then processed the signal. The signal feature was extracted within the frequency domain by using a fast Fourier transform (FFT), and the feature signal was inputted into an artificial neural network (ANN) for training to distinguish between the signals from the normal tool and the worn tool. The results showed that recognition accuracy can reach 76%. The specific process is shown in Figure 3. One study [58] reported that the noise signal acquisition system based on LabVIEW virtual technology can better complete the functions of noise signal acquisition and storage, noise signal filtering, time-domain analysis, and spectrum analysis. Peng et al. [59] used LabVIEW virtual instruments to collect noise signals during milling. The signal and its power spectrum can be observed simultaneously during the acquisition process, and the collected signal can be stored in real-time. The system has a powerful data analysis function, which can provide convenience for subsequent signal analyses and further in-depth research. Figure 4 is the block diagram of sound detection.

### 3.2. Numerical Identification and Analysis of Noise Signals

Digitalization and electronic information technology are developing rapidly at present, and a method for converting acoustic signals into digital signals for processing and analysis is applied in the field of machinery manufacturing. During high-speed cutting machining, the noise generated is composed of irregular combinations of sound waves with different frequencies. Therefore, the analysis of sound signal sources with varying frequency characteristics by using appropriate numerical recognition methods exhibits an important relationship with the prediction of sound signals and the establishment of sound models.

**Figure 5:**
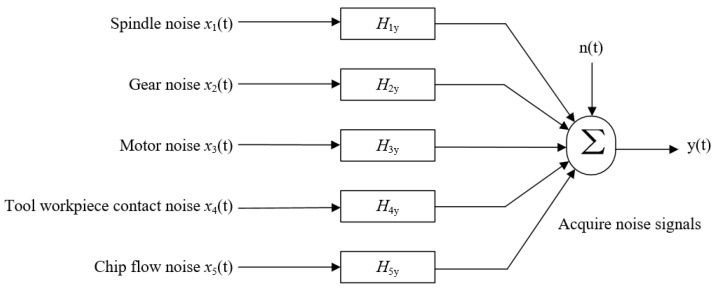
System model.

Wu et al. [60] systematically analyzed the applicable fields of several modern digital signal processing methods. For the multiple-input and single-output noise recognition problem with a correlation between noise sources, the partial coherence analysis method achieves an excellent analysis effect. The mathematical model of the system during actual machining is shown in Figure 5. The blind source separation (BSS) method can effectively separate noise source signals from background noise and echo interference. Moreover, this method is suitable for nonstationary signals with overlapping frequencies. Xue et al. [61] successfully applied the combined wave superposition and BSS method to the sound source identification of mechanical noise signals. This approach has the advantage of requiring a relatively small number of microphones compared to BEM, which requires hundreds (or more) of acoustic sensors. The efficiency of reconstruction can be significantly enhanced. Yang et al. [62] used the BSS method to process the mixed noise signal generated by multiple devices and successfully separated each source signal. The results of simulation signals confirmed the feasibility and effectiveness of the BSS method in noise source separation, which will play an important role in noise identification and signal source identification. But the development of the BSS algorithm depends on original source characteristics, which differ from application to application. Ubhayaratne et al. [63] used a semi-blind signal extraction technique to preprocess and denoise the audio signals collected from stamping operations. They developed an approach to tool wear monitoring in sheet metal stamping using an audio signal processing technique. The results showed that there is a significant qualitative correlation between the audio signals and the tool wear progression during the sheet metal stamping process. More importantly, their research laid the foundation for the use of low-cost audio signal analysis in the development of real-time industrial tool condition monitoring systems. Kothuru et al. [64] developed a tool wear monitoring approach that can classify tool wear conditions using support vector machines (SVM). Audio signals collected during milling processes were transformed into features in the frequency domain. This approach has the advantage of requiring a relatively small number of microphones compared to BEM, which requires hundreds (or more) of acoustic sensors. The efficiency of reconstruction can be significantly enhanced. Cheng et al. [28] proposed a bayesian network, which can effectively integrate sound and current signals and accurately identify the belt wear state. In the case of sufficient training data, the accuracy rate can reach 100%. Portsev and Makarenko [65] used a basic convolutional neural network to process the inputted original data and automatically generate the selection of information features. Lastly, they classified different types of noise signals that can be used to detect the dominant signal in the mixed signals under prior uncertainty conditions. The empirical mode decomposition (EMD) method exhibits high adaptability and nearly does not require setting the parameters manually. This method is suitable for nonstationary signals with an energy ratio and a frequency ratio within a certain range. Based on the IENEMD, the Hilbert transform and 1-D CNNs, Yin et al. [66] established a robust wheel wear detection system for solid carbide grinding under strong noise interference. In IENEMD, a new noise estimation algorithm was proposed to estimate the inherent noise of the raw vibration signals. The repeatable numerical simulations were studied to demonstrate the superior performance of IENEMD. Van et al. [67] adopted a method that combined nonlocal mean denoising with EMD. This method effectively reduced the interference of background noise and other mechanical components. Different from Van’s [67] method, Rubio [68] used independent signal analysis to process the noise generated by a milling machine and the noise generated by the environment, by suppressing them from the signal emitted during the cutting tests and successfully filtered out environmental noise from the collected signals. After graphical analysis and parallel distributed data processing using a supervised neural network (NN) paradigm, the classification of audible sound signal features for process monitoring was obtained. Sun et al. [69] preprocessed the mixed-signal matrix of milling force. Thereafter, they used independent component analysis to process the previous preprocessing results and obtained the independent source signal matrix. Finally, they obtained the spectrum of the independent source signal through FFT and successfully separated and identified high-speed micro-milling force signals, non-Gaussian mechanical noise, and Gaussian environmental noise. In the dynamic detection of acoustic signals, the collected signals are typically superimposed by multiple target signal sources. Independent component analysis based on the independence of signal source separation can effectively separate independent signal sources. During high-speed machining, a noise signal exhibits the characteristics of an unsteady transient signal due to the excitation of the external environment. To extract the characteristics of noise signals effectively, noise signals must be analyzed in the time and frequency domains. Among time–frequency analysis methods, the most commonly used are wavelet transform and short-time Fourier transform. Kwak [70] used wavelet transform and an FFT filter to analyze the extracted original signal quantitatively. The results showed that the image processed via wavelet transform is more similar to the original signal. Noise signal exhibits the characteristics of unpredictability and high destructiveness, and it exerts a considerable negative effect on the processing of the target signal. After capturing audio signals and recording them in Matlab, Prasetyo et al. [57] used Fast Fourier transform to extract signal features in the frequency domain. Then, artificial neural networks were used to classify tool wear. The results showed that the classification performance testing yielded an accuracy of 74%. Yi [71] proved that the wavelet transform method can be used to perform noise smoothing on the target signal with noise. This method improves the reliability of the signal processing results. Tsai et al. [72] proposed an acoustic chatter signal index (ACSI) and the spindle-speed compensation strategy (SSCS) to quantify the microphone signal and regulate the spindle speed. The performance of the real-time chatter prevention strategy based on acoustic signal feedback was determined and verified. Gao et al. [73] used a short-time Fourier transform to calculate the amplitude of nonstationary signals. The calculation process was simple, and the calculation results were accurate and intuitive. This result proved that the short-time Fourier transform exhibits good adaptability to the time–frequency analysis of unsteady signals. A similar study was accomplished by Cao et al. [74]. Four kinds of time-frequency domain analysis methods were involved in the milling sound analysis, including short-time Fourier transform (STFT), continuous wavelet transform (CWT), Wigner–Ville distribution (WVD), and synchrosqueezing transform (SST). Kim [75] used the time–frequency image obtained via short-time Fourier transform as the input signal of an ANN classifier and then applied the neural network classifier to identify different impact noises, distinguishing the actual fault impact noise from the background noise with an accuracy of 100%. He [76] proposed an improved multiwavelet signal processing method with adjacent coefficients and proved that this method can effectively suppress the interference of high-frequency noise on the target signal.

Combined with acoustic theory, the accuracy of the noise source prediction model exerts an important effect on the simulation of a sound field. Djambazov et al. [77] used a coupling algorithm based on the Navier–Stokes equations to simulate the generation of sound waves and airflow characteristics. Moreover, they determined that the algorithm is suitable for establishing an acoustic model of aerodynamic noise generated on a solid surface or in a space that is not reflected and surrounded. Moreover, the method is also applicable to the analysis and calculation of milling cutter aerodynamic noise under high-speed rotation. Sampath et al. [78] developed a cutting noise prediction model that relates cutter–workpiece vibrations to the sound pressure field around the cutter during a high-speed face-milling process. Moreover, they used the model to analyze the effects of various cutting parameters and tool geometric parameters on noise. Previous research [79,80] pointed out that an aerodynamic noise prediction model based on the Navier–Stokes equations for calculating the flow variables of airflow is generally applicable to the analysis and research of the aerodynamic noise of rotating machinery. This finding is consistent with Djambazov’s [77] analysis. Mou et al. [81] reestablished the delay time equation from the perspective of the sound source and transformed it from a transcendental equation to an algebraic equation. This equation can be solved directly without iteration, significantly improving the efficiency of the program to solve an aerodynamic noise field. The flow field solution and sound field solution can be performed simultaneously in the program. Zafar et al. [82] compared and analyzed the signal analysis performance of a backpropagation neural network, a self-organization map, and k-means clustering under four different environmental background noises. The results showed that the backpropagation neural network exhibited the best performance among the three algorithms.

In the field of mechanical machining, noise with different characteristic source signals in the actual machining process must be identified and processed using appropriate methods. This condition is necessary for effective noise control and reduction.

### 3.3. Noise Control and Reduction

Noise is one of the factors contributing to industrial pollution [83]. Noise in machining has attracted the attention of the academic community regardless of whether for ensuring the quality and stability of machining or meeting the current green manufacturing standards. A high noise level can lead to operator discomfort, increased stress, communication problems, and workshop safety problems, and thus, it affects production efficiency. Therefore, predicting and controlling the noise level generated during machining is crucial [78]. The effect of noise on the machining process is primarily reflected in two aspects. On the one hand, the noise exerts an adverse effect on the machining environment. On the other hand, noise signal is mixed as an interference factor in the collected target signal, which significantly influences the extraction and analysis of the target signal.

Identifying the noise source with the largest contribution and optimizing the primary noise source in a targeted and specific manner are important approaches for studying noise control methods. Machine tool noise is one of the primary noise sources in the machining process. In the operation of a positive displacement screw machine, the profile accuracy of the screw rotor has a significant effect on the meshing stability between the male rotor and the female rotor, which is the main factor for noise. Liu et al. [84] studied the influence of installation angle, center distance and grinding wheel wear on rotor profile error and proposed a new method for the prediction profile error of the screw rotor in precision form grinding, which can reduce the noise of machine operation. Mu et al. [85] studied a noise reduction method for the gearing noise of a reinforcing bar straightening cutting machine. Through frequency spectrum analysis, they identified gear noise in a machine tool as the primary noise source. Furthermore, they reduced noise by optimizing the accuracy of the straightening gear structure and improving the assembly quality of the shaft gear. After comparison, the accuracy of the correction gear structure was determined as the most effective in reducing noise. Moreover, the test proved that it can reduce noise to 0.5–2 dB. Yadav and Singh [86] designed the MRGPF tool based on the magnetic flux density distribution on its finishing surface. Further, the experiments were performed to examine the finishing performance of the newly designed tool on the EN-24 steel spur gear teeth profile. The results showed that the surface finishing of gear tooth profile with high shape precision (DIN standard) was improved significantly, and the noise of gear during the operation was reduced.

The tool is an important part of a machining system. Tool structure, tool overhang length and tool wear are considered as noise factors [87,88]. Using the tool as an optimization object is an effective method for reducing noise. Godan et al. [89] analyzed a sound pressure graph and found that noise level depends on the structure of the tool. By coating the tool and optimizing its structure, the radiation of noise to the machining environment can be effectively reduced. Ozbek et al. [90] used coated tungsten carbide tools to turn AISI P20 die steel, the test showed that the PVD TiAlN/TiN-coated tools generated lower noise levels compared to the CVD TiCN/Al_2_O_3_/TiN-coated tools. Pangestu et al. [91] experimentally analyzed the noise level of AlCrN, TiN, and TiAlN coated tungsten carbide in cutting composite boards and found that the coated carbide tools provided lower noise levels for WPC, LVL, and OSB cutting compared to uncoated tungsten carbide cutting tools. Combined with the studies of Godan, Ozbek and Pangestu, we can find that the noise level produced by the coated tool is lower than that produced by the uncoated tool during machining. In addition, Pangestu et al. [92] conducted cutting experiments by using helical edge cutting tools, their results showed that the helical edges provide lower noise emissions compared to the conventional edge (0°), and the noise level decreased with increasing the inclination angle of cutting tool edges. Kvietkova et al. [93] studied the influences of a number of teeth on noise level and edge wear during the sawing process. The results showed that the noise value of the saw blade with less teeth was larger. Li et al. [94] conducted high-speed rotation tests on scribing knives with different structures. After analyzing the noise spectrum of the high-speed rotation of the scribing knife with different groove angles at varying rotating speeds, the noise was determined to reach the minimum value when the groove angle was approximately 91°, 95°, and 105°. Naskrent [95] studied the effect of a number of teeth in the blade of a brush cutter on the noise level. The study found that the noise level decreases as the number of teeth increases. Wang et al. [96] collected the sound pressure signal during the complete turning process and analyzed the spectrum. The analysis indicated that the vibration noise of a high-frequency component of 2–8 kHz is the major body of noise during the turning process. A significant noise reduction effect was achieved through the optimization of the cutter bar damping structure.

Cutting parameters not only determine the machining quality [97], but also play an important role in reducing the sound pressure level of noise and the effect of noise on the processing environment. Rech et al. [98] determined that the stiffness of the part is the most critical parameter that affects noise sound pressure level under the working condition. Moreover, the cutting speed, feed per tooth, and cutting axial depth increase the sound pressure level, whereas radial cutting depth exerts minimal effect on noise. Huang et al. [99] designed a milling noise model by using the axial depth of cut, spindle speed, feed, and radial depth of cut as factors that affect noise. The analysis pointed out that the significance of each factor on milling noise is ordered as follows: axial depth of cut, feed, radial depth of cut, and spindle speed. This conclusion provides ideas for optimizing cutting noise. From the perspectives of energy saving and emission reduction, Zhang et al. [100] established a multi-objective optimization model for energy-saving and noise reduction by optimizing cutting parameters, with cutting speed, feed rate, and cutting depth as decision variables. Tekiner and Yesilyurt [101] recorded processing sound during the turning process by using a computer equipped with a microphone. They determined the optimal cutting parameters in the machining process of AISI 304 stainless steel by considering process sound.

The authors of [102] found that the contact factor between the tool and the workpiece is also an important research direction for noise control methods. Darmawan et al. [103] pointed out that conventional cutting tools with straight edge configurations hit and intermittently contact the workpiece surface during cutting, generating high noise. Hu et al. [104] conducted a milling test on aluminum alloy. After analyzing the noise signal characteristics measured in the time and frequency domains, the vibration between the milling cutter and the workpiece was identified as the primary cause of noise. Arora et al. [105] pointed out that friction noise during turning is determined by the frictional properties of the tool and the workpiece’s surface. Moreover, they found that changing the frictional properties of the tool and the workpiece’s surface by lubricating the tool with oil, coating the tool surface, or using a self-lubricating tool can effectively suppress noise during the machining process.

By accurately identifying the noise source, improving the tool structure, designing reasonable cutting parameters, and optimizing the contact conditions between the tool and the workpiece, it can provide an effective noise control method during high-speed cutting machining.

## 4. Condition Monitoring Based on Noise Signals

Rotating machineries are occupying an increasing position in modern industry and intelligent manufacturing. Real-time monitoring of the working state of rotating machineries cannot only avoid the occurrence of disasters but can also bring evident economic benefits [106,107,108,109]. The process of high-speed machining contains abundant acoustic signals. After eliminating the interference of unwanted noise signals, acoustic signals can return a huge amount of valuable information. For example, tool wear, tool failure, and chatter can be judged by identifying and analyzing acoustic signals. Therefore, acoustic signals can be used as a monitoring index that reflects processing status. In many studies on machining condition monitoring methods, scholars have mostly focused on condition monitoring with the cutting force signal, vibration signal, and acoustic emission signal of non-audible sound frequency as the information carrier. By contrast, relatively few studies have been conducted on the correlation between noise signals within the audible sound range and the machining state.

Monitoring the condition of the tool during the machining process is crucial because the state of the tool directly affects the accuracy and efficiency of workpiece processing. In addition, the continuous operation of a failed tool causes damage to the machine tool. However, most tool monitoring studies have used expensive sensing systems, such as force dynamometers. Even with the use of costly data acquisition hardware, acquiring high-quality data for training the system is difficult and time-consuming because of the complexity of the signals that can be recorded from the operating machine [30]. Research has shown that using acoustic signals as monitoring signals is an important method for improving and developing new tool wear monitoring systems [110,111]. Teti et al. [15] reviewed some papers on the development of a tool wear monitoring system and stated that the tool wear state is related to the sound emitted during machining. It provided a reference for the research of tool wear monitoring by collecting sound signals. Lu and Kannatey [112] used audible sound during the turning process as the basis for tool wear monitoring, analyzed it via FFT, and established a dynamic model for monitoring tool wear state with the sound signal generated during the cutting process. Darmawan et al. [113] applied regression equations to differentiate various stages of tool wear on the basis of the features extracted from the parallel force and noise level. They then tested the feasibility of using noise levels to monitor the degree of tool wear at various cutting speeds. The results of their study showed that the noise level generated by the tested tools was observed to increase linearly with increasing tool wear. Moreover, noise level exhibits a high correlation with tool wear. Raja et al. [114] used Hilbert–Huang transforms to study the relationship between sound and tool flank wear during the turning process. They synthesized and analyzed cutting sound signals under the three states of tool wear (fresh wear, slight wear, and severe wear) and classified wear states with a neural network classifier. The method is simple and reliable, and it can be used in tool flank wear monitoring. Manan et al. [115] used the radial basis function to train a network of features extracted from sound signals and surface roughness to achieve tool condition monitoring. They successfully distinguished among sharp, semi-dull, and dull tools. Lin et al. [26] found through tests that noise increased as the degree of wear increased from the beginning of the tool to the stage of moderate wear of the tool. Moreover, when tool wear reaches a critical value, i.e., near failure, noise signal suddenly changes, and the state of the tool can be judged through this critical value. Madhusudana et al. [24] collected the sound signals of a face milling cutter in healthy and fault states. They extracted a set of discrete wavelet features from the sound signals by using the discrete wavelet transform method. The decision tree (J48 algorithm) technique was used to select prominent features from all the extracted features and input them into the same algorithm for classification. The result can be used for the fault diagnosis of a face milling cutter. Seemuang et al. [30] found that the response of the change of spindle noise power spectrum to the change of cutting speed and feed speed is particularly suitable for tool state monitoring in high-speed machining. As cutting progresses, the tool enters the steady-state wear phase, and the power spectrum of the spindle noise increases rapidly in the latter half of the period until it reaches the maximum value before tool failure suddenly occurs. This sudden turning point is a good indicator of judgment, and it can be used as an early warning signal for tool replacement.

**Figure 6:**
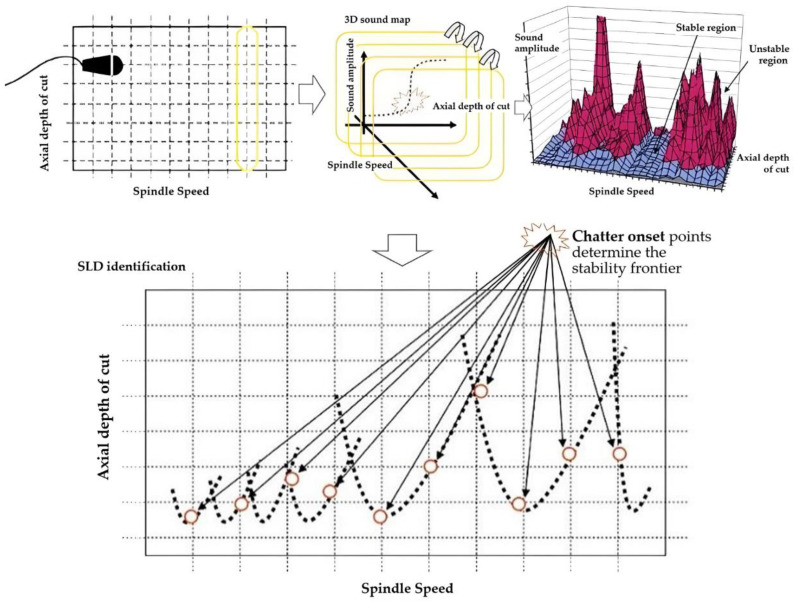
SLD construction methodology.

Chatter is a typical unstable phenomenon in high-speed machining that causes the surface quality of the workpiece to deteriorate and shorten the life span of tools. In severe cases, it causes damage to the tool and even the machine tool, seriously affecting the accuracy and stability of machine tool processing. The acoustic signal is measured using a non-contact measuring device that is convenient and reliable to operate. Studies have shown that chatter monitoring technology based on acoustic signals exhibits a high application value [116]. Delio et al. [117] compared the recognition effects of acoustic signals collected by acceleration sensors, displacement sensors, and microphones on chatter information. The results indicated that the acoustic signal collected by the microphone was more effective for chatter detection. Li et al. [118,119] designed a chatter recognition system for computer numerically controlled machining based on cutting noise testing. This system uses a microphone to collect noise signals and then analyzes and plays back the collected signals through a computer. The test proved that the monitoring method of this system is simple and can accurately determine whether a machine tool is chattering. This system further confirms the study of Delio et al. [117]. Quintana et al. [120] found that the free vibration, forced vibration, and self-excited vibration generated between the material and the workpiece during the milling operation propagated an acoustic signal that contained process information. Chatter frequency can be fed back by constructing a 3D stability lobe diagram (SLD) of sound mapping (as shown in Figure 6) of the collected sound signal to distinguish stable and unstable cutting parameters. The best non-chatter combination of the axial cutting depth and spindle speed can be selected to achieve a higher material removal rate. Altintas [121] performed spectrum analysis on acoustic signals collected during the milling process and found that the maximum amplitude in the spectrum can be used as a detection indicator for chatter.

The acoustic signal is a type of information of the reaction process, and it has a high application value. State monitoring based on acoustic signals provides better feedback with regard to the state of the tool and the stability of processing. Moreover, it does not require complicated equipment and cumbersome operations. Noise acquisition equipment belongs to noncontact equipment. Compared with the acquisition equipment of vibration signal, force signal, and acoustic emission signal, it will not cause interference in the processing, has low acquisition conditions, convenient data analysis, and a short analysis cycle. Therefore, noise signal as the information carrier of the processing state exhibits high practicability and feasibility.

## 5. Conclusions

(1) Noise signal is an acoustic wave signal. The noise signals collected during high-speed machining are formed by combining multiple noise sources, and each noise source has its own characteristic frequency. The target signal after removing the interference noise signal has a high application value. How to accurately classify and identify the target signal in the complex high-speed machining environment is one of the focuses of scholars’ research.

(2) In the high-speed machining process, noise signals are converted into digital signals, and digital signals are numerically identified using computer software. Then, digital signals with different characteristics are processed using appropriate methods. Finally, the cause of noise can be obtained. This process will be of considerable significance to research on noise generation mechanisms and control methods in high-speed material machining.

(3) Accurately analyzing the characteristics of different noise source signals and using appropriate methods for identification and processing are the necessary conditions for effectively controlling and reducing the noise in the process of high-speed cutting. In addition, improving the tool structure, designing reasonable cutting parameters and optimizing the contact conditions between the tool and the workpiece can provide an effective way to control the noise in the process of high-speed cutting.

(4) The collected noise signals in the process of high-speed cutting are mixed with different acoustic signals, which can feedback considerably useful machining condition information, such as tool wear, tool failure, and chatter signals. In addition, compared with the acquisition equipment of vibration signal and force signal, the noise acquisition equipment belongs to non-contact acquisition equipment, which will not cause direct interference to the processing process, and the requirements of acquisition conditions are lower and the data analysis is more convenient. Therefore, the condition monitoring of high-speed machining based on acoustic signals has high practicability and feasibility.

(5) Noise generated during material machining is a result of the coupling of fluid and solid. Further exploring the noise problem through acoustic theory is of considerable value. One is to use the aeroacoustic theory to analyze the effect of the high-speed rotation of the tool on airflow. The other is to use the vibration acoustic theory to analyze vibration problems caused by the dynamic contact between the tool and the workpiece.

## Data Availability

The data presented in this study are available on request from the corresponding author.
